# Trace Metal Requirements and Interactions in *Symbiodinium kawagutii*

**DOI:** 10.3389/fmicb.2018.00142

**Published:** 2018-02-06

**Authors:** Irene B. Rodriguez, Tung-Yuan Ho

**Affiliations:** ^1^Research Center for Environmental Changes, Academia Sinica, Taipei, Taiwan; ^2^Institute of Oceanography, National Taiwan University, Taipei, Taiwan

**Keywords:** trace metals, *Symbiodinium*, photosynthetic endosymbiont, coral bleaching, superoxide dismutase, zooxanthellae

## Abstract

Photosynthetic organisms need trace metals for various biological processes and different groups of microalgae have distinctive obligate necessities due to their respective biochemical requirements and ecological niches. We have previously shown that the dinoflagellate *Symbiodinium kawagutii* requires high concentrations of bioavailable Fe to achieve optimum growth. Here, we further explored the trace metal requirements of *S. kawagutii* with intensive focus on the effect of individual metal and its interaction with other divalent metals. We found that low Zn availability significantly decreases growth rates and results in elevated intracellular Mn, Co, Ni, and Fe quotas in the dinoflagellate. The results highlight the complex interaction among trace metals in *S. kawagutii* and suggest either metal replacement strategy to counter low Zn availability or enhanced uptake of other metals by non-specific divalent metal transporters. In this work, we also examined the Fe requirement of *S. kawagutii* using continuous cultures. We validated that 500 pM of Fe′ was sufficient to support maximum cell density during steady state growth period either at 26 or 28°C. This study shows that growth of *S. kawagutii* was limited by metal availability in the following order, Fe > Zn > Mn > Cu > Ni > Co. The fundamental information obtained for the free-living *Symbiodinium* shall provide insights into how trace metal availability, either from ambient seawater or hosts, affects growth and proliferation of symbiotic dinoflagellates and the interaction between symbiont and their hosts.

## Introduction

Trace metals are required by photosynthetic organisms for key processes including electron and oxygen transport, nutrient acquisition, DNA and RNA production and repair, anti-oxidative defense mechanisms, and many other pertinent biochemical functions (Raven et al., 1999; Sunda, 2012). Marine microalgae acquire bioavailable trace metals from their ambient environment through the action of uptake transporters and by releasing specialized compounds like siderophores or similar machineries (Morel and Price, 2003). The influence of trace metal availability on the growth of some model microalgae, mainly diatoms and coccolithophores, is well documented in literature (e.g., Sunda, 2012). However, there is a knowledge gap about trace metal requirements of endosymbiotic microalgae, such as the genus *Symbiodinium*, which constitutes the largest group of endosymbiotic photosynthetic dinoflagellates found in scleractinian corals and other cnidarians (Pochon et al., 2006; Stat et al., 2008). Owing to its unique ecological niche, the supply of trace metals and other nutrients available to *Symbiodinium* is largely controlled by its host. This entails a complex mechanism to achieve metal homeostasis to maintain critical concentrations of trace metals for necessary biological processes in both hosts and symbionts (Goiran et al., 1996; Rädecker et al., 2015).

In our previous work, we systematically investigated the requirement of *Symbiodinium kawagutii* for trace metal cofactors of superoxide dismutase (SOD) and found that it required the trace metals in the order: Fe >> Cu/Zn/Mn >> Ni (Rodriguez et al., 2016). We observed that the dinoflagellate growth was inhibited in treatments with low Cu/Zn availability and these limiting conditions elevated the quotas of Fe, Mn, and Co, suggesting that there may be functional complementarity between these metals. The absolute importance of Cu and Zn was not distinguished in our prior observations as the two metals were paired together in treatments. Here, batch culture experiments were carried out to evaluate the importance of each individual metal. In addition to Cu and Zn, we also investigated in this study the influence of lack of related divalent metals, such as Mn, Co, or Ni, on *S. kawagutii* growth by varying their concentrations independently or coupled with conditions lacking Cu or Zn. We also further interrogated the importance of Fe availability on dinoflagellate growth using continuous cultures where *S. kawagutii* was kept in steady state growth conditions at different levels of Fe supply. The use of continuous cultures allowed us to investigate the influence of Fe availability on the uptake of other divalent trace metals under the growth temperature of 26°C and at a slightly elevated temperature of 28°C.

In this work, we set out to understand the trace metal requirements of free-living *S. kawagutii* in culture and how the supply of a specific trace metal, e.g., Zn, may influence uptake of other trace metals. Our results may provide information on how bioavailability of these micronutrients affects the growth of free-living dinoflagellates and also shed light on how trace metal supply, which is largely controlled by the host, may dictate the growth of symbiotic photosynthetic organisms.

## Materials and Methods

### General Culture Conditions for Batch Culture Experiments

Non-axenic *Symbiodinium kawagutii* (hereafter referred to as *S. kawagutii*) was obtained from National Center for Marine Algae and Microbiota. The strain, denoted as CCMP2468, was isolated from the scleractinian coral *Montipora verrucosa* and belongs to phylogenetic clade F1. In this work, *S. kawagutii* was grown in trace metal defined medium (Rodriguez et al., 2016) modified from the original L1 recipe (Guillard and Hargraves, 1993). Surface seawater collected from Taiwanese South East Asia Time Series Station (SEATS, 18°N 116°E) was used for culture medium preparation. Seawater was first passed through a column packed with Chelex-100^®^ resin to remove background trace metal contents and filter-sterilized using a 0.22 μm pore size filter prior to use. All batch cultures were carried out in triplicates and were kept in growth chambers with temperature controlled at 26°C. The light was supplied at photon flux density of 680 μmol quanta m^-2^ s^-1^ and operated at a 12:12 h day:night square-wave photoperiod. Initial nitrate and phosphate concentrations were 800 μM and 50 μM, respectively. A mixture of B-vitamins, composed of thiamine, biotin, and cyanocobalamin, was added to culture media to achieve final concentrations of 300, 2.0, and 0.40 nM, respectively. All necessary procedures were carried out in a class-100 trace metal clean laboratory. All materials for culturing and other relevant procedures were washed using 2% Micro-90^®^ solution, rinsed, washed with 10% HCl solution, and rinsed thoroughly with ultrapure water prepared using a Milli-Q system.

### Trace Metal Conditions for Batch Culture Experiments

We carried out two sets of batch culture experiments using 500 ml polycarbonate (PC) bottles. In both sets of experiments, Fe was supplied at 250 nM total dissolved concentration corresponding to an expected inorganic Fe concentration (Fe′) of 1.25 nM upon addition of 20 μM ethylenediaminetetraacetic acid (EDTA). Bioavailability of trace metals in cultures is dictated by the non-chelated or inorganic metal concentration (M′) and this was attained in culture medium by adding EDTA. All of the expected inorganic metal concentrations mentioned in this study were calculated using MineQL version 4.0 (Westall et al., 1976).

We first focused on the interactive effects of Cu, Zn, Mn, and Co on the dinoflagellate growth under higher Cu and lower Mn concentrations compared to levels used in our prior work. We increased the Cu and lowered the Mn concentrations to better simulate natural conditions. We included Co as a metal of interest to investigate interaction between Co, Cu, and Zn due to prior observations that intracellular Co content was elevated in treatments without Cu/Zn (Rodriguez et al., 2016). In this set of experiments, the control cultures contained total dissolved concentrations equivalent to 100 nM for Cu and Zn, and 10 nM for Mn and Co, resulting in expected inorganic metal concentrations of 5.0 pM Cu′, 125 pM Zn′, 4.2 nM Mn′, and 20 pM Co′, respectively. We carried out other treatments where one or a pair of metals was omitted in the culture medium, which was denoted by a minus sign before the element symbol. The summary of trace metal concentrations in different treatments in this set of experiments is presented in **Table 1**.

**Table 1:** Trace metal concentrations in batch cultures to investigate the interactive effects of Cu, Zn, Mn, and Co on *Symbiodinium kawagutii* growth.

Treatment number	Treatment label	Metal availability					
		Cu′ (pM)	Mn′ (nM)	Zn′ (pM)	Co′ (pM)	Fe′ (nM)	Ni′ (pM)
1	Control	5	4.2	125	20	1.25	6.7
2	-Cu	–	4.2	125	20	1.25	6.7
3	-Mn	5	–	125	20	1.25	6.7
4	-Zn	5	4.2	–	20	1.25	6.7
5	-Co	5	4.2	125	–	1.25	6.7
6	+1/10 Cu	0.5	4.2	125	20	1.25	6.7
7	+1/10 Zn	5	4.2	12.5	20	1.25	6.7
8	-Cu/Mn	–	–	125	20	1.25	6.7
9	-Cu/Co	–	4.2	125	–	1.25	6.7
10	-Mn/Zn	5	–	–	20	1.25	6.7
11	-Mn/Co	5	–	125	–	1.25	6.7
12	-Co/Zn	5	4.2	–	–	1.25	6.7


**Control cultures were supplied with trace metals at total dissolved concentrations equivalent to 100 nM for Cu and Zn, 10 nM for Mn, Ni, and Co, and 250 nM for Fe resulting in expected inorganic metal concentrations of 5.0 pM Cu′, 125 pM Zn′, 4.2 nM Mn′, 6.7 pM Ni′, 20 pM Co′, and 1.25 nM Fe′, respectively*.*

We then carried out another experiment to focus on interactive effects of Cu, Zn, and Mn under relatively low Cu and Zn concentrations, which were demonstrated in the previous set of experiments to support growth of *S. kawagutii*. The control cultures were amended with total dissolved concentrations of Cu, Zn, and Mn at 10 nM, corresponding to expected inorganic concentrations of 0.50 pM, 12.5 pM, and 4.2 nM, respectively. We also included additional treatments to validate or further examine the effects of low Fe (50 nM Fe or 250 pM Fe′) and low Zn (2 nM total Zn or 2.5 pM Zn′), which were denoted as +1/5 Fe and +1/5 Zn. The summary of trace metal concentrations in different treatments for this set is presented in **Table 2**.

**Table 2:** Trace metal concentrations in batch cultures to investigate the interactive effects of Cu, Zn, and Mn on *S. kawagutii* growth.

Treatment number	Treatment label	Metal availability					
		Cu′ (pM)	Mn′ (nM)	Zn′ (pM)	Ni′ (pM)	Fe′ (nM)	Co′ (pM)
1	Control	0.5	4.2	12.5	6.7	1.25	20
2	-Cu	–	4.2	12.5	6.7	1.25	20
3	-Mn	0.5	–	12.5	6.7	1.25	20
4	-Zn	0.5	4.2	–	6.7	1.25	20
5	-Cu/Mn	–	–	12.5	6.7	1.25	20
6	-Cu/Zn	–	4.2	–	6.7	1.25	20
7	-Mn/Zn	0.5	–	–	6.7	1.25	20
8	-Ni	0.5	4.2	12.5	–	1.25	20
9	+1/5 Zn	0.5	4.2	2.5	6.7	1.25	20
10	+1/5 Fe	0.5	4.2	12.5	6.7	0.25	20


**The treatments were designed to contain lower Cu and Zn concentrations than in the preceding experiment. Control cultures were supplied with trace metals at total dissolved concentrations equivalent to 10 nM for Cu, Zn, Mn, Ni, and Co, and 250 nM for Fe resulting in expected inorganic metal concentrations of 0.5 pM Cu′, 12.5 pM Zn′, 4.2 nM Mn′, 6.7 pM Ni′, 20 pM Co′, and 1.25 nM Fe′, respectively*.*

### Iron Experiments in Continuous Cultures

To further study the influence of Fe availability on dinoflagellate growth, we kept *S. kawagutii* at steady-state growth using a continuous culture system with trace metal defined medium. We subjected the dinoflagellate to three different total dissolved Fe concentrations equivalent to 50, 100, and 250 nM resulting in 250 pM, 500 pM, and 1.25 nM of Fe′, respectively. The other trace metals were supplied at total concentrations of 10 nM for Cu, Zn, Mn, Ni, and Co resulting in expected inorganic metal concentrations of 0.50 pM Cu′, 12.5 pM Zn′, 4.2 nM Mn′, 6.7 pM Ni′, and 20 pM Co′, respectively. Nitrate, phosphate, vitamin B mixture, and EDTA were supplied at equivalent concentrations as in batch cultures.

For most of the growth period, the cultures were subjected to ambient temperature of 26°C. To elucidate the interactive effects of Fe availability and increase in temperature, the cultures were subjected to an elevated temperature (28°C) for the period covering days 17–23, after which period the temperature was reverted back to 26°C. The cultures were kept in growth chambers with light supplied at photon flux density of 600 μmol quanta m^-2^ s^-1^ operated at a 12:12 h day:night square-wave photoperiod. The system constituted of duplicate 1 L PC magnetic culture vessels fitted with inflow and outflow Teflon tubing maintained at equal rates of 0.270 L day^-1^. The source medium was introduced from a 10 L PC carboy with polypropylene screw cap. The outflow was connected to 500 mL PC containers as algal waste/sampling containers. Continuous stirring of source medium and duplicate culture vessels was done using magnetic stirrers. The rates for both the inflow and outflow were precisely controlled using a multichannel peristaltic pump (IPC 8, Ismatec^®^, Germany). Syringe filters with 0.22 μm pore size were fitted in all containers including source, magnetic culture vessels, and sampling containers to maintain atmospheric pressure equilibrium between the headspace inside the containers and the ambient surroundings.

### Assessment of Growth Rates and Trace Metal Quotas

The growth of *S. kawagutii* was monitored by checking the change in cell density over a period spanning 17 days for batch cultures and 29 days for continuous cultures using a Beckman Coulter Counter Multisizer 3 outfitted with a 100 μm aperture tube. Particle size counting was set within the range 3–8 μm to monitor *S. kawagutii* cells in culture and eliminate possible interference from smaller organisms. There are other methods to monitor changes in cell density but the use of a Coulter Counter offers ease of use combined with excellent precision in measurements that has been demonstrated in dinoflagellates (Krediet et al., 2015). The growth rate in batch cultures was estimated while cells were in exponential phase of growth, typically from days 4 to 10, to ensure minimum contribution from other organisms that may co-exist with *S. kawagutii* in culture. The growth rate in continuous cultures was set at 0.27 day^-1^ during the steady state period of growth using a fixed flow rate of 0.27 L day^-1^ for 1 L culture bottle (Herbert et al., 1956; Dunstan and Menzel, 1971; Touloupakis et al., 2015).

Intracellular trace metal quotas in batch cultures were determined in cells collected during the exponential growth period. In continuous cultures, samples were collected for a total of six times to cover the establishment phase and the steady state growth period. Cells for metal quota determination were collected by filtration onto acid-washed 25 mm PC filters with 2 μm pore size. The cells were quickly rinsed with ultrapure water three times and then acid-digested prior to elemental determination using a high resolution ICPMS (Element XR, Thermo Scientific). Metal quotas were normalized against phosphorus as the biomass indicator (Ho et al., 2003). Procedural filter blanks were subjected to the same digestion, dilution, and analysis. The blank values were subtracted from sample measurements. The detailed information of analytical procedures was described in previous studies (Ho et al., 2003; Ho, 2013).

### Statistical Analyses

One-way analysis of variance (ANOVA) was conducted to determine statistical differences between treatments (*post hoc* Tukey HSD, *p* < 0.05; SPSS).

## Results

### Effect of Trace Metals on *S. kawagutii* Growth

**FIGURE 1:**
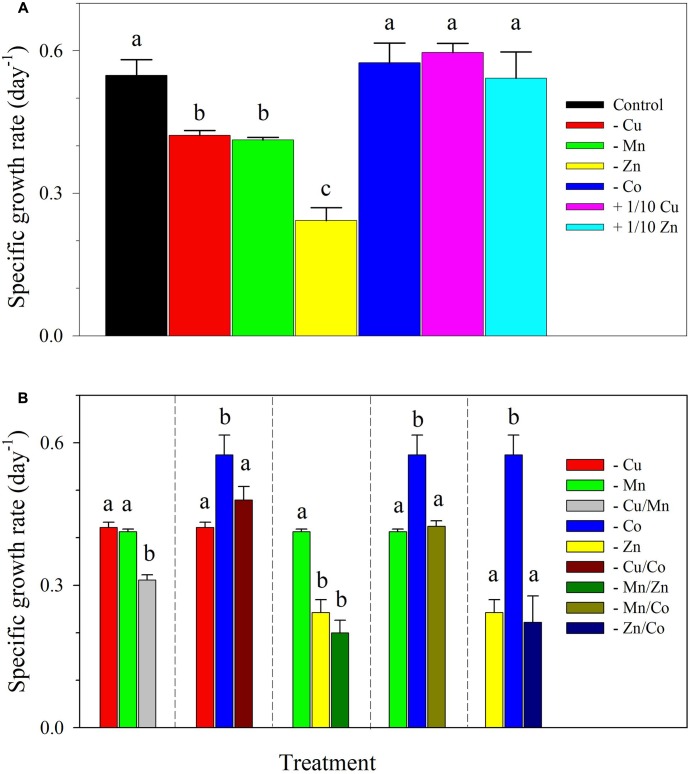
Specific growth rates of *Symbiodinium kawagutii* subjected to different trace metal conditions. **(A)** Independent effect of lack or lower concentration of a metal, and **(B)** interactive effects of lack of a pair of metals. The control treatment was grown with 1.25 nM Fe′, 125 pM Zn′, 5 pM Cu′, 4.2 nM Mn′, and 20 pM Co′. The values represent means ± SD (*N* = 3, *P* < 0.05, ANOVA, *post hoc* Tukey HSD).

The control cultures of *S. kawagutii* achieved a growth rate of 0.55 ± 0.03 day^-1^ and a maximum biomass of about 2.5 × 10^5^ cells ml^-1^ after 14 days of incubation (**Figure 1A** and Supplementary Figure 1). Specific growth rates for treatments showing independent effect of a metal of interest and interactive effects of a pair of metals are presented in **Figure 1**. Among single metal treatments, there was a statistically significant difference between growth rates as determined by one-way ANOVA (*F*_6,14_ = 46.22, *p* < 0.0001). Compared to the growth rate attained by control cultures, the treatment -Co attained a comparable rate of 0.57 ± 0.04 day^-1^. Other treatments also had comparable growth rates to that of the control including the cultures with 10-fold lower Cu or Zn concentrations. The omission of either Cu or Mn resulted in lower rates that were in the same magnitude, 0.42 ± 0.01 day^-1^ and 0.41 ± 0.01 day^-1^, respectively. Among all treatments, the cultures lacking Zn had the lowest growth rate of 0.24 ± 0.03 day^-1^. The effect of lack of a pair of metals on *S. kawagutii* growth is presented in **Figure 1B**. The cultures without Cu/Mn had a growth rate (0.31 ± 0.01 day^-1^) that was significantly lower than in both -Cu and -Mn treatments (*F*_2,6_ = 113.4, *p* < 0.0001). The cultures without Mn/Zn had a rate (0.20 ± 0.03 day^-1^) that was lower than in -Mn treatment but was comparable to the value in -Zn treatment as determined by one-way ANOVA (*F*_2,6_ = 11.62, *p* = 0.009). For all cultures without Co and either one of Cu, Mn, or Zn, the growth rates were comparable to treatments lacking only Cu (*F*_2,6_ = 20.16, *p* = 0.002), Mn (*F*_2,6_ = 25.74, *p* = 0.001) or Zn (*F*_2,6_ = 63.38, *p* < 0.0001), respectively.

**FIGURE 2:**
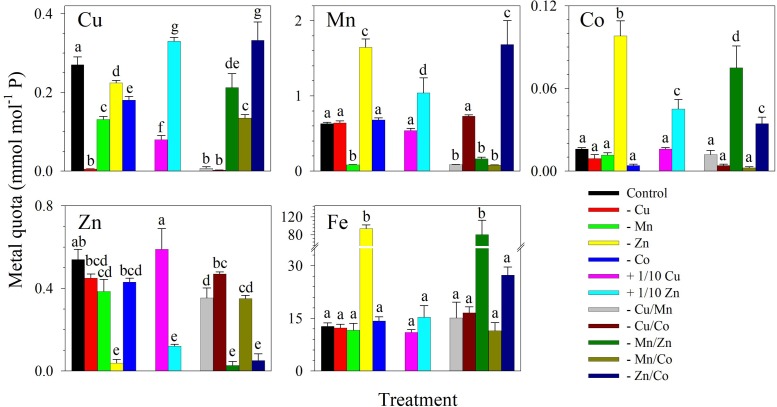
Intracellular trace metal quotas of *S. kawagutii* subjected to different trace metal conditions to investigate the interactive effects of Cu, Zn, Mn, and Co. The control treatment was grown with 1.25 nM Fe′, 125 pM Zn′, 5 pM Cu′, 4.2 nM Mn′, and 20 pM Co′. The values represent means ± SD (*N* = 3, *P* < 0.05, ANOVA, *post hoc* Tukey HSD).

The metal quotas showed that in the control treatment, Fe had the highest intracellular concentration followed by Mn, Zn, Cu, and then Co (**Figure 2**). As expected, intracellular metal quotas were low for metals that were designed to be omitted in culture medium. For example, Zn quotas were lower in treatments lacking Zn including -Zn, -Mn/Zn, and -Zn/Co (*F*_11,48_ = 59.82, *p* < 0.0001) while Mn quotas were lower in cultures without Mn including the treatments -Mn, -Cu/Mn, -Mn/Zn, and -Mn/Co. It was notable that Fe quotas (*F*_11,48_ = 43.83, *p* < 0.0001), as well as Co (*F*_11,48_ = 110.0, *p* < 0.0001) and Mn quotas (*F*_11,48_ = 120.0, *p* < 0.0001) whenever either of these metals was supplied, were significantly elevated in treatments lacking Zn. However, intracellular Fe content was not elevated in the treatment with +1/10 Zn.

**FIGURE 3:**
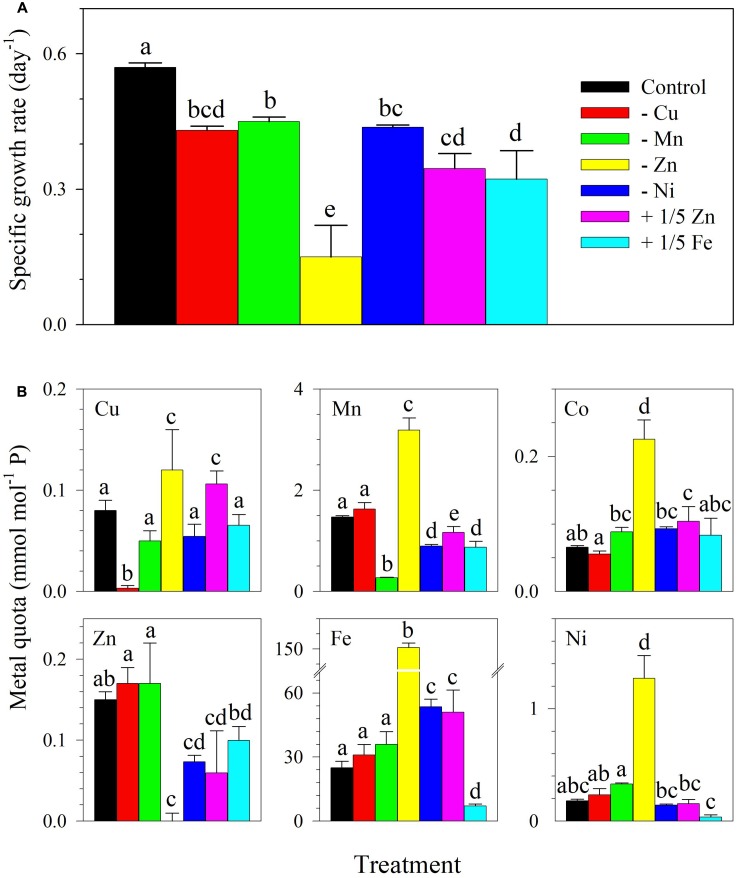
**(A)** Specific growth rates and **(B)** trace metal quotas of *S. kawagutii* subjected to different trace metal availability. The control treatment was grown with 1.25 nM Fe′, 12.5 pM Zn′, 0.50 pM Cu′, 4.2 nM Mn′, 20 pM Co′, and 6.7 pM Ni′. The values represent means ± SD (*N* = 3, *P* < 0.05, ANOVA, *post hoc* Tukey HSD).

In the subsequent experiment, although the control treatment had lower Cu and Zn concentrations, control *S. kawagutii* cultures still attained a growth rate of 0.57 ± 0.01 day^-1^ and reached 8 × 10^5^ cells ml^-1^ after 14 days of incubation (**Figure 3A** and Supplementary Figure 2). There was a statistically significant difference between growth rates among single metal treatments as determined by one-way ANOVA (*F*_6,14_ = 36.70, *p* < 0.0001). It was apparent that cultures lacking Zn had the lowest growth rate of 0.15 ± 0.07 day^-1^ that was significantly different from all other treatments. The treatment with 1/5 Fe had a rate of 0.32 ± 0.06 day^-1^, which was comparable to the treatment with 1/5 Zn (0.35 ± 0.03 day^-1^). Compared with growth rate of control cultures, the treatments -Cu, -Mn, and -Ni all reflected lower rates that were in the range 0.43–0.45 day^-1^. In terms of intracellular content, it was observed again that quotas for specific metals in treatments designed to omit them reflected low values for the metals. For instance, Zn quota was low in the -Zn treatment (*F*_6,28_ = 18.92, *p* < 0.0001) and Cu quota was low in the -Cu treatment (*F*_6,28_ = 25.71, *p* < 0.0001; **Figure 3B**). It was also observed that Mn (*F*_6,28_ = 285.0, *p* < 0.0001), Co (*F*_6,28_ = 65.01, *p* < 0.0001), Ni (*F*_6,28_ = 125.8, *p* < 0.0001), and Fe (*F*_6,28_ = 368.2, *p* < 0.0001) quotas were elevated in the treatment lacking Zn. For treatments elucidating the interactive effects of a pair of metals, the growth curves, rates, and intracellular metal quotas are summarized in Supplementary Figure 3. The growth rates of treatments lacking Zn in combination with either Cu (*F*_2,6_ = 27.18, *p* = 0.001) or Mn (*F*_2,6_ = 34.31, *p* = 0.001) were comparable to the rate observed in the treatment lacking Zn.

### Influence of Fe Availability on *S. kawagutii* Growth in Continuous Cultures

**FIGURE 4:**
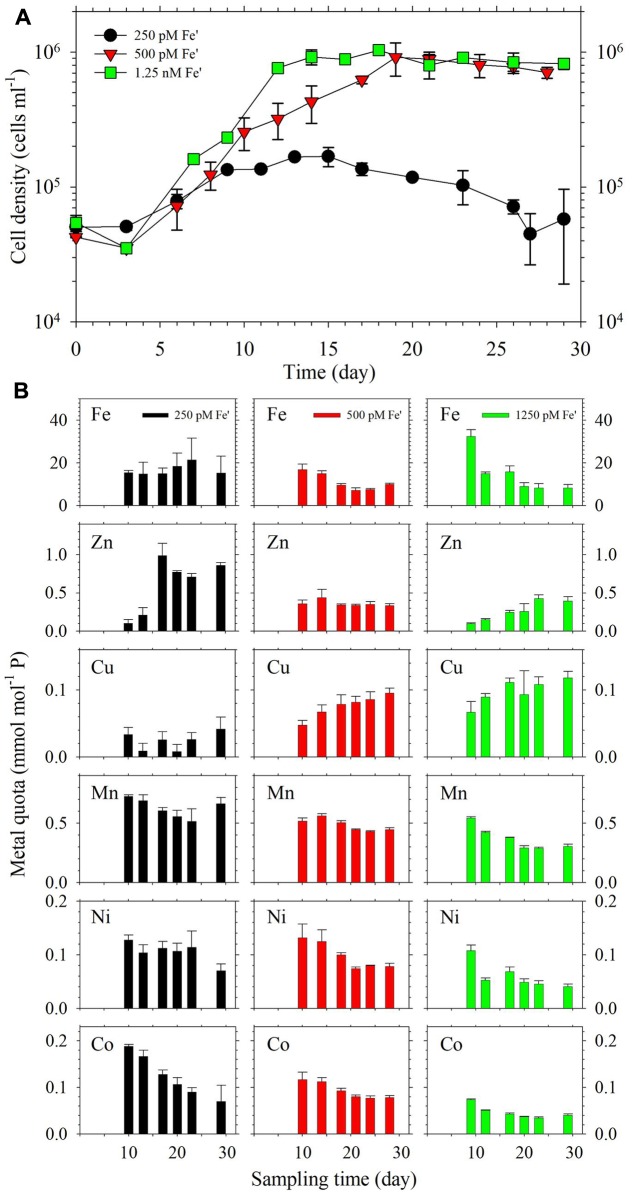
**(A)** Growth curves and **(B)** intracellular metal quotas of *S. kawagutii* subjected to different Fe availability in continuous culture conditions. The other trace metals were supplied at the following concentrations: 12.5 pM Zn′, 0.50 pM Cu′, 4.2 nM Mn′, 20 pM Co′, and 6.7 pM Ni′. Error bars represent average deviation of duplicate cultures.

The growth curves of the dinoflagellate in continuous cultures are presented in **Figure 4A**. In terms of cell density, the results show that 1.25 nM Fe′ supported high cell density at the level of 10^6^ cells ml^-1^ during the steady state growth period, which was attained after about 12 days. The cultures supplied with 500 pM Fe′ also achieved comparable cell density after 19 days while the cultures amended with 250 pM Fe′ only reached about 2 × 10^5^ cells ml^-1^. The increase in growth temperature from 26 to 28°C for the period covering days 17–23 did not result in observable changes in cell density for cultures subjected to 500 pM or 1.25 nM Fe′. Intracellular metal quotas in cells were evaluated at different times during the incubation period (**Figure 4B**). Fe quotas in cultures with 250 pM Fe′ were within the range 14.9–15.4 mmol mol^-1^ P during the steady state growth period from days 10 to 17. In cultures with 500 pM Fe′, the Fe quotas were within the range 7.1–10.1 mmol mol^-1^ P for the period covering days 18–28. The cultures with 1.25 nM Fe′ showed the longest steady state period from days 12 to 29 wherein Fe quotas were observed at 15.0 and 15.8 mmol mol^-1^ P before stabilizing in the range 8.4–9.1 mmol mol^-1^ P. There was significant increase in Zn quotas corresponding to the period of temperature change in the treatment with 250 pM Fe′, which were not observed in cultures provided with higher Fe availability. Mn quotas varied slightly among different treatments, with higher values observed in the treatment with 250 pM Fe′ ranging from 0.51 to 0.73 mmol mol^-1^ P compared to 0.43–0.56 and 0.29–0.54 mmol mol^-1^ P in cultures supplied with 500 and 1,250 pM Fe′, respectively. An increasing trend was observed for Cu quotas in treatments with higher Fe availability, albeit changes were not significant for most. For Ni quotas, relatively lower values were observed in cultures with 1.25 nM Fe′ compared to that in 250 and 500 pM Fe′ cultures. Co quotas showed higher values in 250 pM Fe′ cultures compared to the two treatments with relatively high Fe′.

## Discussion

### Effect of Trace Metal Availability on *S. kawagutii* Growth

In our previous work on *S. kawagutii*, we coupled Cu and Zn together in treatments due to their coexistence in SOD. The coupled effect of Cu and Zn on the dinoflagellate growth was significant but it was difficult to distinguish their individual importance (Rodriguez et al., 2016). By varying the availability of Cu or Zn individually, the results of this study demonstrate that Zn has a stronger influence on the dinoflagellate growth compared to Cu. This was evidenced by lower growth rates observed in -Zn treatments than in -Cu treatments, either independently or in combination with lack of either Mn or Co (**Figures 1**, **3** and Supplementary Figure 3). These results indicate that the reduced growth observed in the -Cu/Zn treatment in our previous work (Rodriguez et al., 2016) and observed again here was mainly due to Zn limitation. The results also place Zn after Fe and make it the second most needed trace metal by *S. kawagutii*. The essential role of Zn is expected for dinoflagellates because Zn is a cofactor in carbonic anhydrase, alkaline phosphatase, RNA polymerase, reverse transcriptase, and other enzymes (Raven et al., 1999; Sunda, 2012). In addition to -Zn treatments, we also evaluated the minimum Zn concentration that will support maximum growth of *S. kawagutii* by growing the cells with 12.5 pM Zn′, one tenth of the concentration used in the control (+1/10 Zn, **Table 1** and **Figure 1A**). The growth rate observed in +1/10 Zn treatment was comparable to that in control cultures, indicating that 12.5 pM Zn′ was sufficient for optimal growth. In brief, our results show that Zn deficiency may negatively affect the dinoflagellate growth and highlight the need to investigate bioavailable Zn concentrations in ambient seawater surrounding coral reef areas, as well as understand the mechanism for Zn uptake and trafficking within the coral holobiont.

Copper may serve as a micronutrient or a toxic agent for microalgae, depending on its bioavailable concentration in seawater (Festa and Thiele, 2011). Indeed, an early report has established the negative effects of Cu on marine dinoflagellates and posited that high Cu concentrations were probably arresting cell division or causing change in cell metabolism (Saifullah, 1978). Also, elevated levels of labile Cu have been shown to impede growth of the dinoflagellates *Amphidinium carterae* and *Prorocentrum micans* as well as reduce protein production in the latter (Lage et al., 1994). On the other side of its toxic role, Cu is pertinent in the action of transcriptional regulators, oxidoreductases, and in chaperones or storage (Festa and Thiele, 2011). Total dissolved Cu concentrations can reach to 45 nM in riverine and submarine groundwater discharge near populous regions (Donat et al., 1994). Cu is also highly abundant and soluble in anthropogenic aerosols, which may be transported to offshore coral reef areas seasonally (Sholkovitz et al., 2010). We deemed it necessary to elucidate the optimum range of Cu supply that permits dinoflagellate growth. The inorganic Cu concentration used in our previous work was relatively low, 0.50 pM, compared to inorganic concentrations of Fe, Mn, and Zn, which were 50–1,250 pM, 42 nM, and 125 pM, respectively (Rodriguez et al., 2016). In this study, we initially increased Cu′ to 5.0 pM and also carried out a treatment with 10-fold lower Cu′ (**Table 1** and **Figure 1A**). Our results clearly indicate that 5 pM Cu′ did not inhibit growth of *S. kawagutii* and that 0.50 pM Cu′ was sufficient to support favorable growth of the dinoflagellate (**Figure 1A**). When grown in -Cu conditions, with potential carry-over Cu from the inoculum, the dinoflagellate was still able to attain high cell density albeit at a much slower growth rate. These results indicate that the dinoflagellate has an obligate need for Cu that may be met even with low Cu availability.

Manganese is mainly utilized in the oxygen-evolving complex of PSII as well as in maintenance of chloroplast structure in photosynthetic organisms. A study on *Amphidinium* sp. found that Mn availability was positively correlated with growth rate, maximal photochemical efficiency, and Mn-SOD production of the dinoflagellate (Cao et al., 2011). Our previous study shows that 42 nM Mn′ was sufficient for *S. kawagutii* to reach maximum growth. In this study, we used 4.2 nM Mn′ in the control treatment, one tenth of the concentrations used in our previous work to better simulate the Mn concentrations in the natural environment (Rodriguez et al., 2016). The results show that 4.2 nM Mn′ was sufficient to support *S. kawagutii* growth. Thus, we used this concentration in subsequent set of experiments. Mn exhibits relatively high dissolved concentrations in oceanic surface water, with dissolved concentrations generally ranging from 2 to 10 nM (Landing and Bruland, 1987). It is thus unlikely that Mn would become a limiting factor for *S. kawagutii* growth in natural environments. In this work, the dinoflagellate was also grown under lack of both Cu and Mn in the -Cu/Mn treatment (**Figure 1B** and Supplementary Figure 3). In both instances, the growth rate attained by the -Cu/Mn treatment was always lower than in both the -Cu and -Mn treatments indicating a synergistic negative effect of lack of both metals on *S. kawagutii* growth. This observation certainly warrants further study.

We also carried out a treatment lacking Ni and the result showed that lack of Ni resulted in significantly lower growth rate than in control cultures (0.43 ± 0.01, *p* = 0.001, **Figure 3A**). This finding was contradictory with our prior observation that Ni only slightly affects the growth of the dinoflagellate (Rodriguez et al., 2016). The disparity in results may have been due to interactive effects of trace metals supplied at specific concentrations. In our prior work, Zn, Mn, and Ni were supplied at 10-fold higher concentrations compared to levels used in this set of treatments (**Table 2**). The relatively high concentrations of trace metals used in our prior work may have fully or partially replaced the requirement for a specific metal in treatments, e.g., in -Ni conditions (Rodriguez et al., 2016). In terms of Co requirement, we carried out four treatments lacking Co independently or paired with Zn, Cu, or Mn (**Table 1** and **Figure 1**). For the independent effect of -Co, we did not observe significant influence on *S. kawagutii* growth. When paired with other metals, the results show that the growth was more affected by availability of the other metal rather than Co. For instance, growth of -Mn/Co cultures was similar to the growth performance of -Mn cultures (**Figure 1B**). These results suggest that Co was required at low concentrations or it may not be essential for *S. kawagutii* growth. Overall, by compiling the results obtained from both sets of experiments, trace metal requirements of *S. kawagutii* follows the order: Fe > Zn > Mn > Cu > Ni > Co. Trace metal concentrations in the environment may be influenced by various processes including natural and anthropogenic activities with the latter largely viewed as a major factor determining biogeochemical cycling of trace metals. This entails that trace metal concentrations may have a stronger influence on microalgae inhabiting inshore ecosystems rather than organisms thriving in the open ocean or offshore ecosystems.

### Effect of Fe Availability on *S. kawagutii* Growth in Continuous Cultures

In our previous work, we have established that *S. kawagutii* requires 500 pM Fe′ to sustain high growth rates under batch culture conditions (Rodriguez et al., 2016). Although batch cultures offer an efficient and convenient way of studying the interactive effects of trace metals on algal growth, the diminishing availability of major nutrients and vitamins during the incubation period also influence trace metal requirements. Continuous cultures represent a better simulation of natural conditions in terms of mimicking the conditions of micronutrient and major nutrient supplies (Lippemeier et al., 2001). The use of continuous cultures also allows maintenance of microalgae in a steady state growth condition wherein the growth rate is regulated by controlling the flow rates of both inflow of fresh medium and outflow of culture medium. Prior to the steady state growth period, which is called the establishment phase, algal cells in continuous culture grow in similar performance as that in batch culture and growth rates may be higher than both flow rates (Gresham and Hong, 2014). Once in steady state growth period, the growth rate of algal cells is equivalent to the flow rate, which ideally should be equivalent for both inflow and outflow, divided by the volume of the growth medium (Herbert et al., 1956; Touloupakis et al., 2015). To ensure that we only observe the influence of Fe availability, we carried out the continuous culture experiments by only varying Fe′ under exactly same flow rates and all other growth conditions were identical. Our results validated our previous observation that 500 pM Fe′ supports optimum growth of *S. kawagutii* as demonstrated by high cell density attained in this treatment when cultures reached steady state condition (**Figure 4A**). The cultures with 1.25 nM Fe′ attained comparable cell densities to cultures with 500 pM Fe′ and reached steady state growth a week earlier, indicating that higher Fe supply leads to a shorter establishment phase of *S. kawagutii*. The cultures with 250 pM Fe′ achieved steady state condition at lower cell density, suggesting that insufficient Fe concentration inhibited the proliferation of *S. kawagutii*. Endosymbionts in the coral *Acropora millepora* have been reported to reach densities of 1.4 × 10^6^ cells cm^-2^, which corresponded to a *Symbiodinium* to host cell ratio of 15 to 100 (Mieog et al., 2009). The observed cell densities in treatments with higher Fe indicate that symbiotic dinoflagellates may require high bioavailable Fe concentrations to sustain high cell densities in intact holobionts. This highlights the need to study the Fe trafficking, and also that of other metals, within the coral holobiont.

The growth of the dinoflagellate in continuous culture was also studied under a slight increase in growth temperature, which was done from days 17 to 23 by rapidly increasing the temperature to 28°C, an increase of 2°C from the ambient temperature. It has been reported that prolonged exposure to temperatures that are 2°C higher than the average monthly maximum may lead to coral bleaching (Jokiel and Coles, 1990). Although the exposure to higher temperature only lasted for 1 week, this may offer insights into how free-living dinoflagellate responds to a sudden increase in temperature. In this study, we observed that 500 pM and 1.25 nM Fe′ conditions were able to sustain the dinoflagellate growth during the temperature change. We propose that sufficient Fe supply sustains growth of *S. kawagutii* during sudden pulses of temperature increases. This has important implications in the resilience of host-dinoflagellate associations because Fe availability within the holobiont may influence the capability of *Symbiodinium* to cope with temperature stress by producing Fe-containing anti-oxidative enzymes.

### Metal–Metal Interaction in *S. kawagutii*

Intracellular metal quotas of *S. kawagutii* show that the dinoflagellate takes up high concentrations of Fe, followed by Mn, Zn, Cu, Ni, and Co in the control treatment (**Figure 3B**). In this set of treatments with lower Cu and Zn concentrations, Zn and Cu quotas were comparable in cultures supplied with both metals. These results suggest that intracellular uptake for both metals were equivalent despite the dissimilarity of their effect on *S. kawagutii* growth (**Figure 3A**). In treatments lacking Zn, independently or in combination with other metals, we observed the significant elevation of intracellular quotas of Fe, Mn, Ni, and Co, particularly Fe (**Figures 2**, **3B** and Supplementary Figure 3C). The elevation in quotas of other divalent metals was also observed in the low Cu/Zn treatment in our previous study (Rodriguez et al., 2016). Our results demonstrate that this increase was largely due to Zn limitation as there were no elevated intracellular quotas observed in treatments lacking Cu. Further examination of results indicates that increase in quotas was indeed ascribed to Zn limitation. For example, Fe and Mn quotas were elevated in -Zn and -Zn/Co but not in -Co cultures, and Fe and Ni quotas were elevated in -Zn and -Mn/Zn but not in -Mn cultures. It is tempting to attribute the increase in quotas of divalent cations to activity of non-specific metal transporters (Lane et al., 2008). However, the increase may also be caused by biological requirement or metal replacement in specific biological processes. For instance, the elevated Fe and Mn quotas in low Zn conditions may be due to increased production of Fe- or Mn-containing enzymes required in pertinent photosynthetic systems and anti-oxidative defense networks (Sunda, 2012). Also, the observed elevation in Co quotas may be related to activity of carbonic anhydrase. Among Zn-requiring metalloproteins, carbonic anhydrase, the enzyme that catalyzes the reversible reaction between bicarbonate and free CO_2_, is arguably the most important (Sunda, 2012). The metal cofactor of carbonic anhydrase in diatoms may be replaced by either Co or Cd (Price and Morel, 1990; Sunda and Huntsman, 1995). The elevated Co quotas in low Zn conditions hint at the possibility of Co replacement for Zn, which may be beneficial for *S. kawagutii* especially in Zn limited conditions. Typical Zn concentrations in the surface water of the open ocean are relatively low, generally ranging from 0.3 to 2 nM and its bioavailability is further reduced by strong complexation with naturally occurring organic ligands (Bruland, 1989; Crawford et al., 2003). Future studies are needed to examine bioavailable Zn concentrations and evaluate the importance of Zn as a limiting factor for *Symbiodinium* in coral reef areas.

In the continuous culture study, the metal quotas show that intracellular Fe quotas were slightly higher in the treatment with 250 pM Fe′ than in treatments with 500 pM and 1.25 nM Fe′ despite having lower cell density throughout the incubation period. The higher intracellular Fe content may be attributed to reduced growth dilution because of lower cell density in the treatment with 250 pM Fe′ (Sunda and Huntsman, 1998). Among all of the metal quotas determined, intracellular Mn quotas exhibited the least variation among the three treatments while Ni and Co quotas were slightly higher in cultures with 250 pM Fe′ than in the other two treatments. The elevation in Ni and Co quotas may be related to uptake of non-specific divalent metal transporters when Fe is limited in culture medium (Lane et al., 2008). In addition, Cu quotas were lowest in cultures with 250 pM Fe′. The interactive effects of Cu and Fe on phytoplankton growth and succession have been studied in diverse organisms (Maldonado et al., 2006; Semeniuk et al., 2016). There are at least two biological roles associated with Cu including the involvement of Cu in high-affinity Fe transport systems of some diatoms and the use of Cu-containing plastocycanin instead of Fe-containing cytochrome c_6_ in *Thalassiosira oceanica* (Maldonado et al., 2006; Peers and Price, 2006). Our results show that low Fe availability did not elicit an increase in Cu quotas, indicating that Fe–Cu interaction in *S. kawagutii* may be owed to other biological processes or uptake exclusion by same transporters. The last and most notable trend was pertaining to Zn quotas in cultures supplied with 250 pM Fe′, which reflected higher values during the period of higher growth temperature. Cu/Zn-SOD is an important anti-oxidative enzyme. Increase in Zn quotas may be attributed to increased requirement for Cu/Zn-SOD at higher growth temperature. However, corresponding Cu quotas were not elevated, which underscores the need to include complementary determination of protein level information with intracellular metal content in future studies. For instance, parallel information on SOD expression may shed light on how the dinoflagellate utilizes various SODs when it is grown in media with different availability of metal cofactors.

## Conclusion

The growth rates and intracellular metal quotas observed in this study provide an insight on the trace metal requirements and metal interactions in *S. kawagutii*. In this work, we have elucidated the effect of different trace metals, including Fe, Cu, Zn, Mn, Co, and Ni, on the growth of *S. kawagutii* and established the relative intracellular requirements of the dinoflagellate. In addition, we demonstrated the use of continuous cultures to elucidate the influence of Fe availability and study metal–metal interaction in *S. kawagutii*. Our results offer distinctive and useful primary information into the complex and interconnected processes utilizing metals in free-living *Symbiodinium*. Also, these results may be valuable to infer on the trace metal requirements of symbiotic dinoflagellates existing within the holobiont where supply of trace metals and other nutrients depend on the host. For future studies on free-living or symbiotic *Symbiodinium* species, it will be beneficial to obtain parallel information about metal-containing proteins and genes expression with their growth rates and intracellular metal content for a better understanding of the biochemical functions of trace metals in *Symbiodinium*.

## Author Contributions

IR and T-YH designed the study, analyzed the data, and wrote the manuscript; IR performed the experiments.

## Conflict of Interest Statement

The authors declare that the research was conducted in the absence of any commercial or financial relationships that could be construed as a potential conflict of interest.
