# Supplementary material for: Trace Metal Requirements and Interactions in Symbiodinium kawagutii
Source: Front Microbiol. 2018 Feb 6;9:142. doi: 10.3389/fmicb.2018.00142 (PMC5808119; doi:10.3389/fmicb.2018.00142)
Supplement: Supplementary file 1 [file Data_Sheet_1.docx]

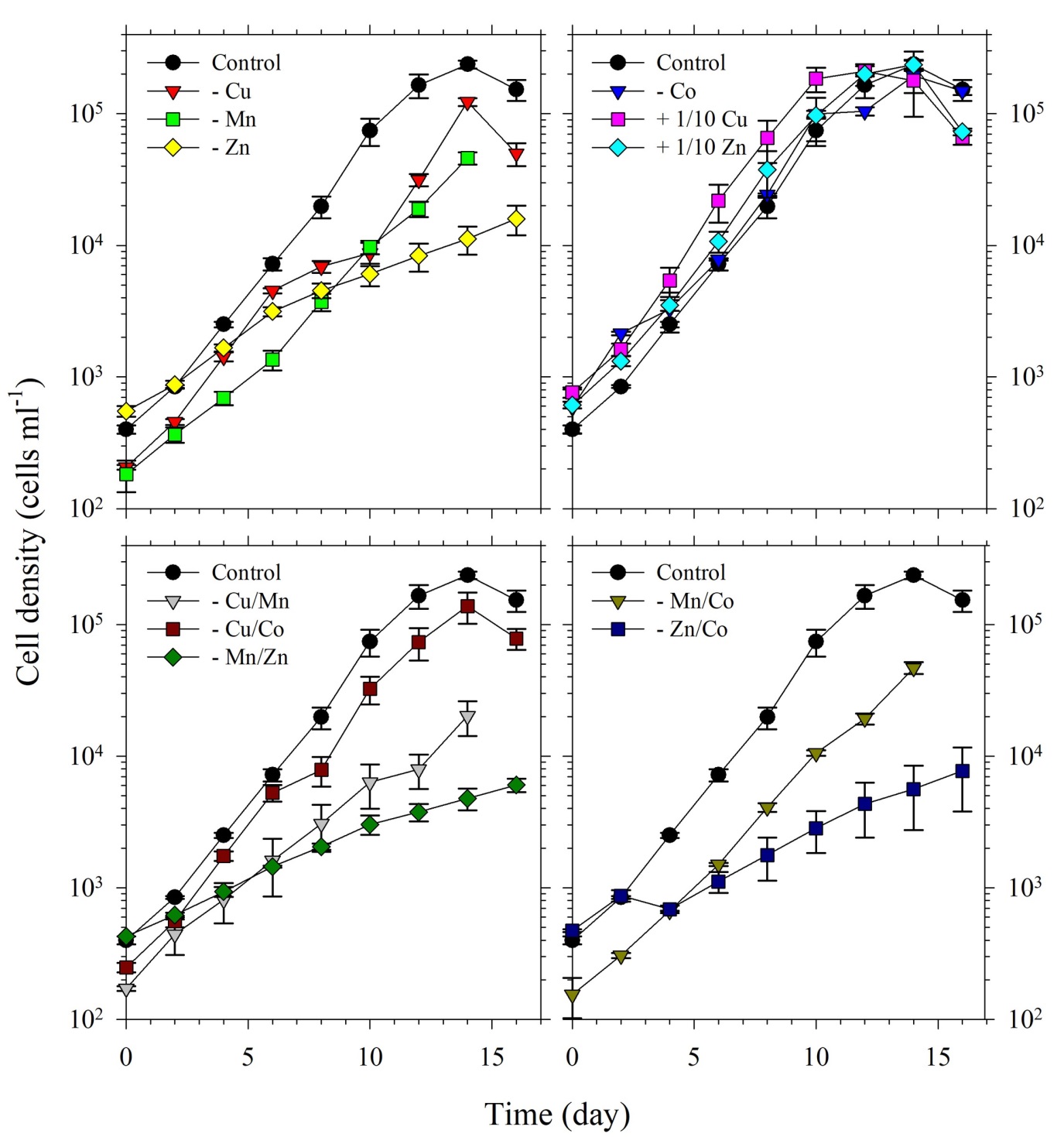


**Supplementary Figure 1.** **Growth curves of *S. kawagutii* subjected to different trace metal conditions to study the interactive effects of Cu, Mn, Zn, and Co.** The control treatment was grown with 1.25 nM Fe′, 125 pM Zn′, 5 pM Cu′, 4.2 nM Mn′, and 20 pM Co′. Error bars represent standard deviation of triplicate cultures. The growth rate in batch cultures was estimated while cells were in exponential phase of growth, typically from days 4-10.


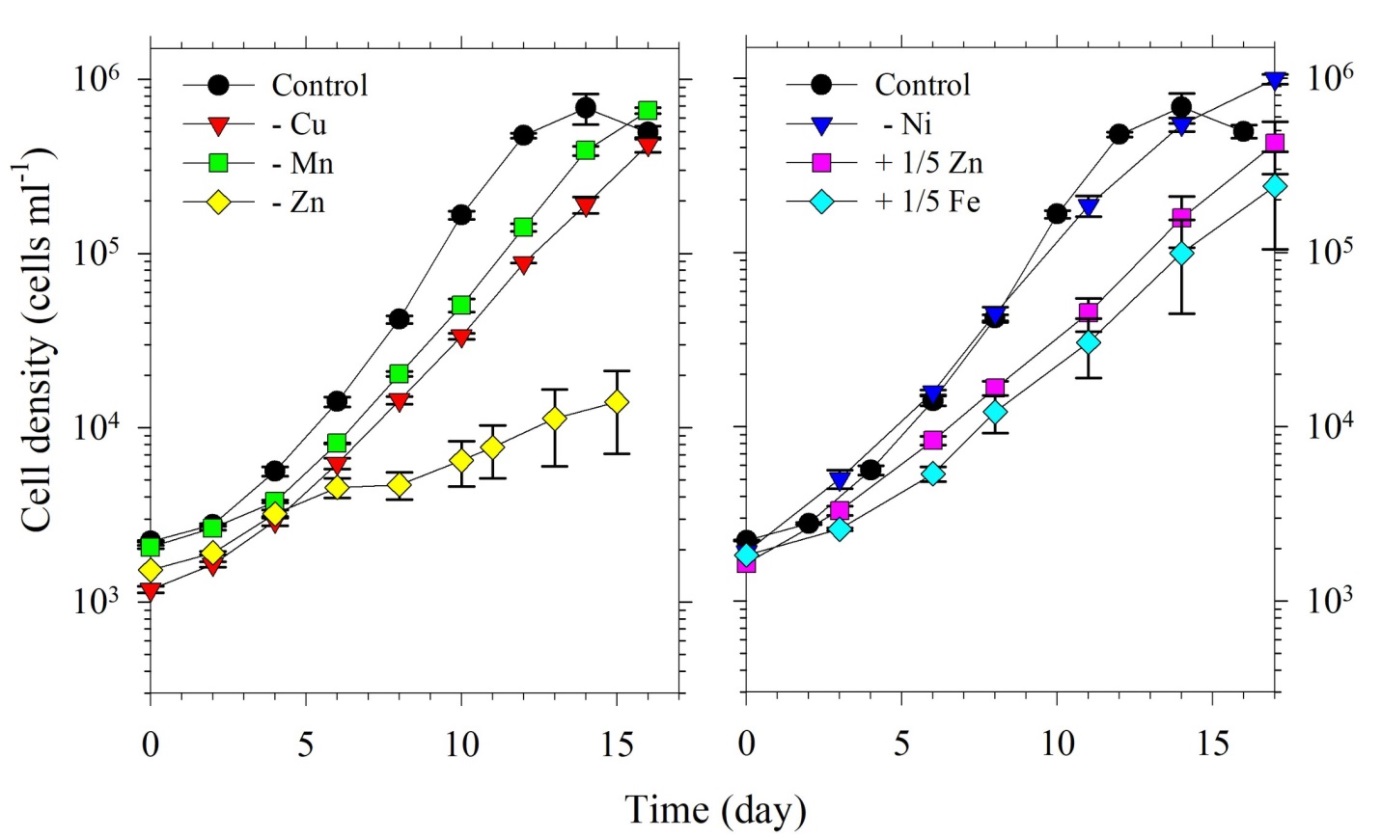


**Supplementary Figure 2.** **Growth curves of *S. kawagutii* subjected to different trace metal availability.** The control treatment was grown with 1.25 nM Fe′, 12.5 pM Zn′, 0.50 pM Cu′, 4.2 nM Mn′, 20 pM Co′, and 6.7 pM Ni′. Error bars represent standard deviation of triplicate cultures. The growth rate in batch cultures was estimated while cells were in exponential phase of growth, typically from days 4-10.


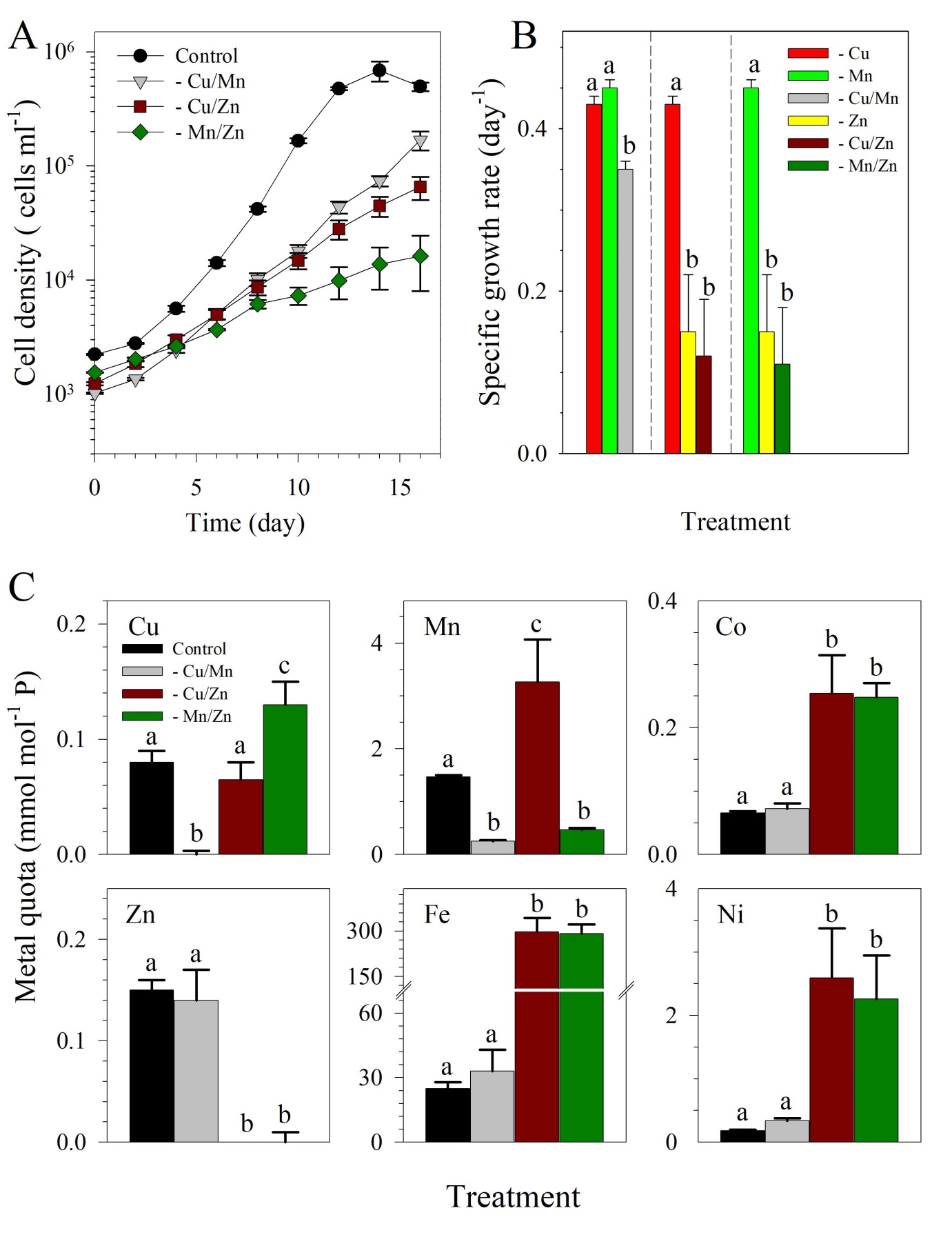


**Supplementary Figure 3.** **Growth curves and intracellular metal quotas of *S. kawagutii* subjected to different trace metal availability to study the interactive effects of Cu, Zn, and Mn.** The control treatment was grown with 1.25 nM Fe′, 12.5 pM Zn′, 0.50 pM Cu′, 4.2 nM Mn′, 20 pM Co′, and 6.7 pM Ni′. The values represent means ± SD (*N* = 3, *p* < 0.05, ANOVA, *post hoc* Tukey HSD).The growth rate in batch cultures was estimated while cells were in exponential phase of growth, typically from days 4-10.
